# Digital mental health apps and the therapeutic alliance: initial review

**DOI:** 10.1192/bjo.2018.86

**Published:** 2019-01-29

**Authors:** Philip Henson, Hannah Wisniewski, Chris Hollis, Matcheri Keshavan, John Torous

**Affiliations:** Research Assistant, Division of Digital Psychiatry, Beth Israel Deaconess Medical Center, Harvard Medical School, USA; Research Assistant, Division of Digital Psychiatry, Beth Israel Deaconess Medical Center, Harvard Medical School, USA; Chair of Child and Adolescent Psychiatry and Director, NIHR MindTech MedTech Co-operative, NIHR Nottingham BRC Mental Health; and Technology Theme Lead, University of Nottingham and Institute of Mental Health, UK; Stanley Cobb Professor and Vice-Chair for Public Psychiatry, Department of Psychiatry, Beth Israel Deaconess Medical Center, Harvard Medical School, USA; Director of Digital Psychiatry, Departments of Psychiatry and Clinical Informatics, Beth Israel Deaconess Medical Center, Harvard Medical School, USA

**Keywords:** Smartphone, alliance, Individual Psychotherapy

## Abstract

**Background:**

As mental healthcare expands to smartphone apps and other technologies that may offer therapeutic interventions without a therapist involved, it is important to assess the impact of non-traditional therapeutic relationships.

**Aims:**

To determine if there were any meaningful data regarding the digital therapeutic alliance in smartphone interventions for serious mental illnesses.

**Method:**

A literature search was conducted in four databases (PubMed, PsycINFO, Embase and Web of Science).

**Results:**

There were five studies that discuss the therapeutic alliance when a mobile application intervention is involved in therapy. However, in none of the studies was the digital therapeutic alliance the primary outcome. The studies looked at different mental health conditions, had different duration of technology use and used different methods for assessing the therapeutic alliance.

**Conclusions:**

Assessing and optimising the digital therapeutic alliance holds the potential to make tools such as smartphone apps more effective and improve adherence to their use. However, the heterogeneous nature of the five studies we identified make it challenging to draw conclusions at this time. A measure is required to evaluate the digital therapeutic alliance.

Among the 44.7 million adults living with mental illness in the USA, only 43.1% received mental health treatment in the prior year.^1^ As technology attempts to bridge the healthcare access gap, it is increasingly important that quality of treatment remains both high and consistent. One element of such high-quality care is a strong therapeutic relationship, defined as the working alliance between the patient and therapist that is composed of shared goals, agreement with tasks and development of a bond.^2^ Generalisable to all psychotherapies, the therapeutic alliance that is established during face-to-face therapy is considered predictive of positive outcomes^3^ and essential to medical care.^4^

## Changes in the delivery of therapy and the impact on the therapeutic alliance

The introduction of computers into therapy and clinical visits has brought new challenges for providers including balancing the management of the electronic health record with establishing a meaningful relationship with the patient.^5^ Although the introduction of technology may in some cases lead to overall improvements in quality and efficiency of care,^6^ an inverse relationship exists between clinician computer use and the quality of the therapeutic alliance.^3^

More recently, researchers have begun to investigate the usability and efficacy of smartphone interventions in mental health as the popularity of mobile applications (apps) continues to expand. Although smartphone apps have the potential to aid people with depression,^7^ bipolar disorder^8^ and suicide ideation,^9^ identifying quality, evidence-based apps still remains a challenge. People who seek support for their well-being not only use health apps on a daily basis,^10^ but also have over 10 000 mental health apps to choose from that are available from commercial marketplaces.^11^ As more patients turn to,^12^ or are referred to mental health apps, it is important to understand the efficacy of these apps^13^ and the impact of the frequent lack of human support in their use.^14^ Therapists recognise that the unsupervised use of apps is concerning,^15^ and that there is a need to understand the nature of the therapeutic relationship that patients have with these apps, which we will refer to in this article as the digital therapeutic alliance.

Initial attempts to address this question have focused on the impact of computer and internet interventions on a patient–online therapist alliance, suggesting online therapy may generate a similar therapeutic relationship to that in face-to-face therapies,^16^ but there is little consensus as to how to measure the digital therapeutic alliance for smartphone apps and mobile devices.^17^ One study modified a validated scale, the Working Alliance Inventory (WAI), by changing some of the language, for example replacing ‘therapist’ with ‘online therapist,’ but the authors noted the limitation of using a scale originally designed for face-to-face therapy.^18^ Both patients and clinicians seek clarity on this topic with the digital therapeutic alliance being voted as a top ten research priority in a recent national study involving 600 mental health stakeholders in the UK.^19^

## Aims of our literature review

Given the importance of the therapeutic alliance in therapy, the scalability of mobile phone interventions and the popularity of mental health apps, we conducted a literature search to determine if there were any meaningful data regarding the digital therapeutic alliance in smartphone interventions for serious mental illness. We realise that our results may be limited because we have only included studies that involve smartphones, but we determined that it was critical to establish a baseline in order to know where the field currently stands regarding the digital therapeutic alliance in mental health apps.

## Method

### Search strategy

A literature search was conducted in four databases (PubMed, PsycINFO, Embase and Web of Science) on 19 July 2018. The specific search terms (see Appendix 1) were generated with the help of a librarian and included the mention of alliance (or support, bond, relationship) in the title or abstract, a serious mental illness and a mobile app or smartphone. The search resulted in 2501 abstracts. An additional study was identified through other sources and added to the search results.

### Selection strategy

Duplicates were removed to yield 1507 abstracts, which were subsequently screened by two reviewers (P.H. and H.W.) using the software Rayyan^20^ with the following inclusion criteria: (a) individuals are at risk for or are diagnosed with a serious mental illness (such as depression, bipolar disorder, schizophrenia or schizoaffective disorder); (b) individuals are interacting with the software outside of a laboratory environment; and (c) therapeutic alliance (or support, bond, relationship) is mentioned in the title or abstract.

The exclusion criteria were: (a) in a foreign language; (b) reviews or study protocols; and (c) software was not smartphone-focused. One study was replaced by another from the same authors that more properly fitted our criteria but was not returned in the search results. All conflicts were discussed with a third reviewer (J.T.) until consensus was reached.

**Fig. 1:**
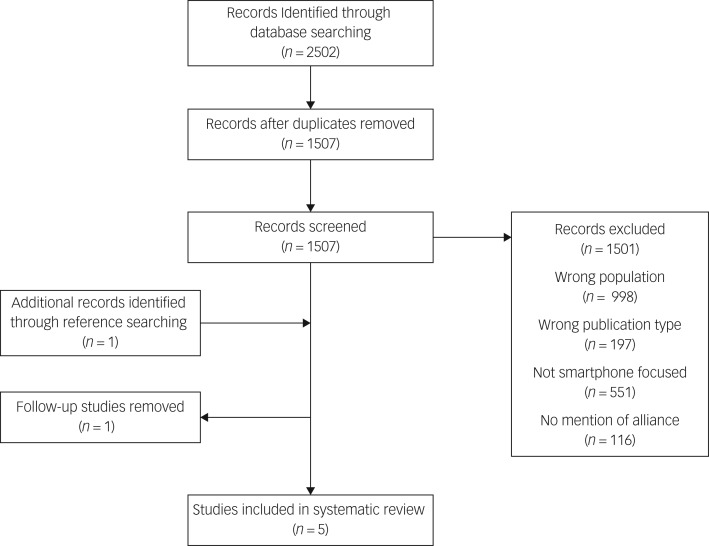
PRISMA 2009 flow diagram.

This strategy resulted in five studies that discuss the therapeutic alliance when a mobile application intervention is involved in therapy (see Fig. 1 for the PRISMA 2009 flow diagram^21^ for our study).

### Data extraction

Study characteristics were obtained from the five studies and included: (a) authors name(s); (b) diagnosis of patients enrolled in study; (c) sample size and mean age of patients; (d) intervention used and length of treatment; (d) outcome(s) measured; (e) therapeutic alliance scale used; and (f) study quality, which was evaluated using a 27-item checklist designed for assessing both randomised and non-randomised studies.^22^

## Results

Basic study characteristics and quality scores are outlined in Table 1.

**Table 1:** Study characteristics

Authors	Sample size and age	Patient condition	Intervention and length of treatment	Outcome(s) measured	Therapeutic alliance scale used	Study quality^22^
Forchuk *et al*^23^	*n* = 41; age: mean 17.0 years, s.d. = 1.4	Depressive symptoms	Lawson SMART record, 6 months	Usability, acceptability	None; evaluated qualitatively via thematic analysis	21/32
Richards & Simpson^24^	*n* = 7; age: mean 27.5 years, s.d. = 7.8	Major depressive disorder, persistent depressive disorder; health anxiety disorder; social anxiety disorder; borderline personality disorder; bulimia nervosa	goACT web and mobile-based with cognitive– behavioural therapy; mindfulness-based cognitive therapy; dialectical behavioural therapy; 20 weeks	Therapeutic alliance, therapeutic engagement, distress	Agnew Relationship Measure – Short Form	24/32
Mackie *et al*^25^	*n* = 6; age: mean 27 years, s.d. = 8	Men presenting with self-harm	ACHESS^a^ mobile health application with problem-solving therapy; 6–10 weeks	Effectiveness, engagement, depression (secondary)	None; evaluated qualitatively via thematic analysis	15/32
Reid *et al*^26^	*n* = 68; age: mean 18.1 years, s.d. = 3.2	Mild or more severe emotional/mental health issue, Kessler Psychological Distress Scale symptom score >16	MTYPE^b^ program; daily for 2–4 weeks	Depression/anxiety/stress,^28^ clinician usability, doctor–patient rapport, pathways to care	General Practice Assessment Questionnaire Communication and Enablement subscales, Trust in Physician Scale	28/32
Bauer *et al*^27^	*n* = 17; age: 18–65+ years	Depressive disorder, anxiety disorder	Ginger.io; 3–4 times per week for 4–8 weeks	Depression, anxiety, usability, acceptability, satisfaction	None; evaluated qualitatively via thematic analysis	22/32

a. Addiction Comprehensive Health Enhancement Support System.

b. Mobile Tracking Young People's Experiences.

Forchuck *et al*^23^ investigated usability and acceptability of a smartphone accessible electronic personal health record (ePHR) system called Lawson SMART record (LSR) among young people with depressive symptoms and found via thematic analysis the potential for the LSR to enhance the patient–therapist alliance.

Richard & Simpson^24^ investigated the effects of a web-based technology (goACT) as an adjunct to psychotherapy. They used a short form of the Agnew Relationship Measure (ARM-5) and did not find significant changes in the therapeutic alliance between therapist and patient before and after using goACT.

Mackie *et al*^25^ studied the effectiveness of a smartphone application (ACHESS) originally designed to treat harmful substance use in men presenting with self-harm who were veterans. They found via thematic analysis that the therapeutic alliance with technology is related to app engagement and a function of trust and communication.

Reid *et al*^26^ monitored young people with emotional mental health issues using a mobile phone application. The control group used a similar version of the mobile phone application that did not contain mental health questions. The therapeutic alliance between the patient and physician was measured via the General Practice Assessment Questionnaire and the Trust in Physician Scale and the authors found no major differences between the experimental and control groups.

Bauer *et al*^27^ monitored depression and anxiety symptoms using the Ginger.io smartphone-based platform. They found via thematic analysis that the care manager's relationship with the patient affected the feasibility and acceptability of a mobile health platform adjunct.

## Discussion

### Main findings

The lack of studies exploring the impact of mobile technology on the therapeutic relationship is surprising given the potential impact the therapeutic relationship may have on the efficacy of smartphone and digital-based interventions. The heterogeneous nature of the five studies we identified, including differences in individuals' mental health condition, duration of technology use and method of therapeutic alliance assessment, make it challenging to draw conclusions at this time.

### Interpretation of our findings

Of the five studies, two used scales to measure the therapeutic alliance,^24^^,^^26^ and only one alludes to a digital therapeutic alliance.^25^ None of the studies attempted to quantify the digital therapeutic alliance. Although all papers sought to evaluate the feasibility and acceptability of a new mobile tool for mental health intervention, the therapeutic alliance was never the primary outcome or central concern. In Mackie *et al*,^25^ the interviewer asks one participant if he felt supported by the app ‘during times that you were away, not directly in the face-to-face therapy’. The participant replies, ‘It was a non-issue. It [the app] didn't give me any security because it didn't work’. This case of faulty technology leading to a lack of security suggests that the digital therapeutic alliance in part many depend on good app design and functionality. Yet the digital therapeutic alliance is not purely technical and understanding the interplay between smartphone app features like privacy and safety, efficacy, engagement and data sharing^29^ along with patient's personal and clinical goals remains the challenge.

In general, the studies seemed to agree that smartphones as an adjunct to therapy can lead to increased engagement and adherence,^23^^,^^25^^,^^27^ and that key factors in the therapeutic alliance involve being able to communicate and share information with a clinician outside the normal therapy window.^23^^–^^25^^,^^27^ The question still remains how the relationship may change with varying degrees of clinician interaction, and what constitutes the key factors of the therapeutic alliance when interventions are delivered solely by smartphone. The lack of any literature on this latter point is notable given the rapid expansion of apps directed towards self-guided treatments.^30^

Today's smartphone apps that often attempt to translate cognitive–behavioural therapy manuals into smartphone formats have yielded less impressive results than expected^31^ and a better understanding of the digital therapeutic alliance may offer a solution to increase efficacy and better realise the true potential of digital mental health.

### Scales for measuring the digital therapeutic alliance

The lack of studies reporting on the digital therapeutic alliance may also be in part a result of the lack of any practical scale to assess a digital therapeutic alliance. Simply replacing ‘therapist’ with ‘online therapist’ on alliance scales such as the WAI is limited in that important factors in face-to-face therapies may not be equally important in internet-based interventions.^18^ Berry *et al* has started to bridge that gap by introducing a new version of the ARM for digital health interventions,^32^ but more research and consensus must be developed to test the implementation of these new measurements. At this point it remains unclear if measurements of the digital therapeutic alliance will be universal or whether it will be necessary to customise them by region, culture, age and technology literacy.

A research agenda exploring patients' relationships to their own smartphones, the use of smartphones in clinical care and the use of smartphone apps for self-care is necessary and urgent given the rapid expansion of mobile technology for care. We propose the use of a simple, easy to implement scale for measuring the therapeutic alliance based on the successful validation of a short form of the WAI (WAI-SR).^33^ The Digital Working Alliance Inventory (D-WAI, see Appendix 2) would concisely assess the same core factors as WAI-SR – goals, tasks and bond – to quickly measure the therapeutic relationship between patient and app. Future research is required to test the D-WAI in a real-world setting.

Previous systematic reviews of the therapeutic alliance in face-to-face therapy have found positive relationships between alliance and outcome but in general studies focused on the patient–therapist alliance rather than the patient–software alliance, and included a broader search of internet-delivered interventions.^17^^,^^34^

### Limitations

It should be mentioned that our study has several weaknesses. The nature of our search terms may have limited our results to studies that discuss the therapeutic alliance but do not highlight it in their paper. In addition, our search terms could have been modified to include words such as ‘psychotherapy’, ‘psychology’ or ‘counseling’ which are relevant components to the therapeutic alliance and likely would have aided the screening process.

We focused on smartphones interventions because of the scalability of distribution for mobile apps, but in doing so may have missed insightful analyses on the digital therapeutic alliance with other internet-delivered interventions. This research also raises a fundamental question about the nature of the therapeutic relationship as an agent of change in the next generation of digital interventions using artificial intelligence, ‘chatbots,’ and ‘virtual human’ therapists.^19^^,^^35^ These genuinely interactive interventions attempt to humanise the therapy experience and may present challenges in evaluating the digital therapeutic alliance.

In conclusion, as innovative technology is adapted to fit healthcare needs it is important that the focus expands from product development to care delivery.^36^ Among the many mental health apps available for download, many of them place responsibility on mental well-being in the hands of the patient.^30^ Thus, assessing and optimising the digital therapeutic relationship holds the potential to make tools like smartphone apps more effective and improve adherence to their use.

## Appendix 1

### Search terms for literature review

PubMed: (alliance[TIAB] OR relationship[TIAB] OR bond[TIAB] OR support[TIAB]) AND (mental health OR psychiatry OR bipolar disorder OR schizophrenia OR schizoaffective OR depression) AND (‘Mobile Applications’[Mesh] OR ‘Cell Phone’[mesh] OR ‘Computers, Handheld’[mesh] OR ((app OR apps OR application* OR technology OR software) AND (phone[tiab] OR phones OR handheld* OR mobile)) OR smartphone* OR mobile device* OR handheld device* OR iphone* OR ipad* OR android*).

PsycInfo: (mental health OR psychiatry OR bipolar disorder OR schizophrenia OR schizoaffective OR depression) AND (‘Mobile Applications’ OR ‘Cell Phone’ OR ‘Handheld Computers’ OR ((app OR apps OR application* OR technology OR software) AND (TI phone OR AB phone OR phones OR handheld* OR mobile)) OR smartphone* OR mobile device* OR handheld device* OR iphone* OR ipad* OR android*)) AND ((TI alliance OR AB alliance OR TI relationship OR AB relationship OR TI bond OR AB bond OR TI support OR AB support).

Embase: (‘mental health’/exp OR ‘mental health’ OR (mental AND (‘health’/exp OR health)) OR ‘psychiatry’/exp OR psychiatry OR ‘schizophrenia’/exp OR schizophrenia OR ‘schizoaffective’/exp OR schizoaffective OR ‘depression’/exp OR depression) AND ((‘mobile applications’/exp OR ‘mobile applications’ OR ‘cell phone’/exp OR ‘cell phone’ OR ‘handheld computers’ OR ((appOR apps OR application* OR ‘technology’/exp OR technology OR ‘software’/exp OR software) AND (‘phone’:ab,ti OR phones OR handheld* OR mobile)) OR ‘smartphone* OR mobile’ OR (smartphone*or AND mobile AND device*) OR handheld) AND device* OR iphone* OR ipad* OR android*) AND (‘alliance’:ab,ti OR ‘relationship’:ab,ti OR ‘bond’:ab,ti OR ‘support’:ab,ti).

Web of Science: (TS = ((alliance OR relationship OR bond OR support) AND (mental health OR psychiatry OR bipolar disorder OR schizophrenia OR schizoaffective OR depression) AND (‘Mobile Applications’ OR ‘Cell Phone’ OR ‘Handheld Computers’ OR ((app OR apps OR application* OR technology OR software) AND (phone OR phones OR handheld* OR mobile)) OR smartphone* OR mobile device* OR handheld device* OR iphone* OR ipad* OR android*))).

## Appendix 2

### Digital Working Alliance Inventory (D-WAI)

1. I trust the app to guide me towards my personal goals [Goals]
2. I believe the app tasks will help me to address my problem [Tasks]
3. The app encourages me to accomplish tasks and make progress [Bond]
4. I agree that the tasks within the app are important for my goals [Goals]
5. The app is easy to use and operate [Tasks]
6. The app supports me to overcome challenges [Bond]

Goals: 1 and 4

Tasks: 2 and 5

Bond: 3 and 6
